# Cellular Senescence as a Risk Factor in Head and Neck Cancer—Diagnostic and Therapeutic Perspective

**DOI:** 10.3390/cancers18010087

**Published:** 2025-12-27

**Authors:** Magdalena Stachowiak, Magdalena Kostrzewa, Wojciech Golusinski, Pawel Golusinski, Ewelina Golusinska-Kardach, Michal M. Masternak, Błażej Rubiś

**Affiliations:** 1 Department of Head and Neck Surgery, Poznan University of Medical Sciences, 61-701 Poznan, Poland; wgolus@ump.edu.pl (W.G.); michal.masternak@ucf.edu (M.M.M.); 2 Department of Clinical Chemistry and Molecular Diagnostics, Poznan University of Medical Sciences, 60-806 Poznan, Poland; 87114@student.ump.edu.pl (M.K.); blazejr@ump.edu.pl (B.R.); 3 Doctoral School, Poznan University of Medical Sciences, 60-812 Poznan, Poland; 4 Department of Otolaryngology and Maxillofacial Surgery, University of Zielona Gora, 65-417 Zielona Gora, Poland; pgolusinski@uz.zgora.pl; 5 Department of Dental Surgery, Periodontology and Oral Mucosa Diseases, Poznan University of Medical Sciences, 60-812 Poznan, Poland; ekardach@ump.edu.pl; 6 Burnett School of Biomedical Sciences, College of Medicine, University of Central Florida, Orlando, FL 32827, USA

**Keywords:** senescence, head and neck cancer, HNC diagnostics, HNC therapy, HNC risk factors

## Abstract

Head and neck cancer (HNC) affects nearly 1 million people every year. The main risk factors include tobacco, alcohol, or human papilloma virus (HPV) viral infections, which contribute to HNC development through various, mostly unknown, mechanisms. One of these postulated mechanisms is cellular senescence. This biological aging-associated process is responsible not only for the arrest of cellular growth and division but also mediates the modulation of cell metabolism and secretory phenotype. Consequently, it may play a crucial role in carcinogenesis, which makes it an interesting topic in the context of cancer development, prognosis, prevention, and therapy. This review focuses on the current state of knowledge regarding all aspects of the association between cellular senescence and head and neck cancer.

## Simple Summary

Head and neck cancer (HNC) affects nearly 1 million people every year. While the risk factors are widely known (e.g., tobacco, alcohol, or human papilloma virus (HPV) infections), the mechanisms underlying them are not yet fully understood. One of the postulated mechanisms associated with HNC is cellular senescence, a process triggered by stress and aging. The primary characteristic of senescent cells is the secretion of various factors, collectively referred to as the senescence-associated secretory phenotype (SASP). It leads to various responses of the organism and contributes to carcinogenesis. In this review, we compile current information on senescence mechanisms in HNC development and senescence-associated cancer therapies. This article presents a new perspective on the approach to HNC treatment and suggests a way for further studies on the subject.

## 1. Introduction

Head and neck cancer (HNC) affects regions in the upper aerodigestive tract, with the highest incidence in the lip and oral cavity (41% of HNC), larynx (20%), nasopharynx (13%), oropharynx (11%), hypopharynx (9%), and salivary glands (6%) [1]. It is responsible for over 940,000 new cancer cases worldwide, which accounts for 4.7% of total cancer cases and over 480,000 deaths yearly (14.9% of all cancers), ranking HNC in sixth place when compared to other types of cancers [1]. Notably, the data showed that the incidence of HNC and mortality is higher in men than in women, indicating sex as a significant variable in this disease [1]. There are many subtypes of HNC, but the most common one, squamous cell carcinoma, accounts for over 90% of HNC cases [2]. Significantly, numerous factors contribute to the increased risk of HNC development, and some of the mechanisms are [3] or may be mediated by pathways associated with senescence. Even though there are many reviews discussing cellular senescence in cancer, the novelty of our work is its specific focus on the role of senescence in head and neck cancer (HNC).

## 2. HNC Risk Factors

**Figure 1:**
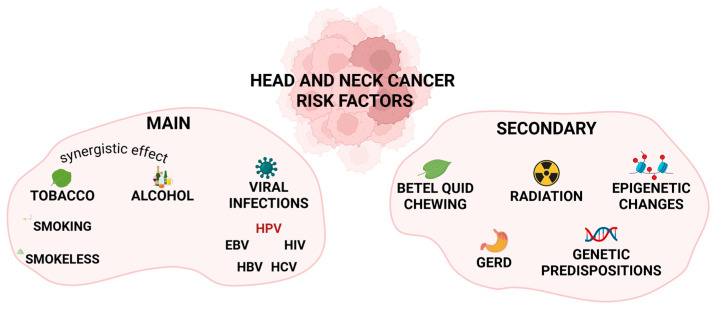
Main and secondary risk factors for head and neck cancer including tobacco, alcohol, and viral infections, as well as betel quid use, radiation exposure, GERD, genetic predispositions, and epigenetic changes. Main risk factors have a greater influence on head and neck cancer formation than secondary factors. HPV—human papilloma virus, EBV—Epstein–Barr virus, HIV—human immunodeficiency virus, HBV—hepatitis B virus, HCV—hepatitis C virus, GERD—gastroesophageal reflux disease. Created in BioRender. Rubis, B. (2025) https://BioRender.com/2lgnjan (accessed on 12 December 2025).

Several risk factors contribute to HNC development, with tobacco, alcohol, and HPV infections being the most common determinants (Figure 1). There are two different groups of tobacco products-intended for smoking (e.g., cigarettes, cigars, bidi, chutta, kretek, but also water pipes) and in the smokeless form (e.g., chewing tobacco, snuff, dry powdered tobacco, topical tobacco paste, or gutka) [4,5]. All the mentioned forms of tobacco intake show some carcinogenic effects. The studies show that smokers are around twice as likely to develop head and neck squamous cell carcinoma (HNSCC) when compared to people who have never smoked. While quitting decreases the risk, it takes approximately 10 years to reduce the risk by half and around 20 years to lower it by over 80% [4,6,7]. Combustible tobacco products contribute more significantly to HNSCC than smokeless products, with cigarettes posing the highest cancer risk among all tobacco types [4]. However, chewing tobacco significantly increases the risk of oral cancer [8]. Tobacco smoke contains over 70 compounds associated with oncogenic potential, and smokeless tobacco comprises over 30 of them. Among those carcinogens, there are two most commonly investigated groups, i.e., tobacco-specific nitrosamines (TSNAs) and polycyclic aromatic hydrocarbons (PAHs) [4,5,8]. TSNAs in combustible tobacco are a group of nitrosamines consisting of seven compounds. On the other hand, in smokeless tobacco, the most harmful compounds from the TSNAs are 4-(methylnitrosamino)-1-(3-pyridyl)-1-butanone (NNK) and N′-nitrosonornicotine (NNN) [5].

Moreover, smokeless tobacco contains heavy metals such as cadmium and lead, polonium, and formaldehyde, all of which are classified as carcinogens [5,8]. These compounds cause head and neck cancer (HNC) through metabolic activation by cytochrome P450 enzymes, which generate local chronic inflammation, oxidative stress, and reactive species formation. All of them can lead to carcinogenesis via DNA damage, causing genetic instability [4,8]. This causes permanent mutations leading to oncogene activation (e.g., K-ras) or tumor suppressor inactivation (e.g., p53), resulting in uncontrolled cell proliferation.

In vitro studies show that both nicotine-containing and nicotine-free e-cigarette liquids cause oxidative stress, leading to oxidative DNA damage [9,10]. The effect depends on the dosage and exposure duration [9,10,11]. Some tested liquids increased TNF-α, MMP3, IL8, and IL1αin OSCC (oral squamous cell carcinoma) [12,13]. Research on normal and HNSCC cell lines revealed DNA strand breaks and death of the cells caused by e-cigarettes [14]. E-cigarette smokers have reduced function of genes and pathways related to HNSCC, such as NOTCH1 and HERC2 [15]. E-cigarettes not only contribute to cancer formation but also decrease the therapeutic effect of cisplatin during chemotherapy [16].

Interestingly, e-cigarettes were presented as a safer alternative to regular cigarettes. However, vape liquids are composed of a wide range of ingredients, making their safety assessment difficult. However, more and more studies have appeared showing a negative impact of e-cigarettes on human health and their possible association with cancer formation [17].

Another risk factor, alcohol consumption, directly influences HNC formation through its metabolites, i.e., mainly acetaldehyde and reactive oxygen species (ROS) [18,19]. Additionally, some of the fermentation products formed during alcohol production reveal carcinogenic effects [18]. Each year after quitting alcohol, the risk of alcohol-related HNC decreases by 2% [20]. HNC risk increases with the dose, but it is also higher for those who drink moderately but regularly [21]. There are several pathways of alcohol-related HNC carcinogenesis. Similar to tobacco carcinogens and their metabolites, alcohol carcinogens and their metabolites cause HNC by forming DNA covalent adducts [18]. This leads to mutations, tumor suppressor genes inactivation, and oncogene activation, ultimately resulting in cancer growth [18]. One of those adducts is acetaldehyde, the primary metabolite of alcohol, formed in the oxidation process by alcohol dehydrogenase (ADH). Additionally, acetaldehyde disrupts DNA synthesis and repair, binds to enzymes (e.g., glutathione, O6-methyl-guanylyltransferase), and contributes to mucosa lesions development [18,19]. Increased cytochrome P450 2E1 (CYP2E1) induces ROS formation and decreases retinol and retinoic acid levels that impair cell growth and differentiation [18,19]. Chronic alcohol consumption causes mitochondrial dysfunction that also induces ROS generation [18]. Moreover, some oral bacteria and yeasts have the ability to convert ethanol into acetaldehyde, significantly increasing acetaldehyde concentration in saliva (10–100 times higher than in blood), causing local acetaldehyde exposure [18,19,22,23]. In addition, ethanol increases those microorganisms’ levels, leading to greater acetaldehyde formation. Simultaneously, it lowers the anti-inflammatory and antioxidant action by reducing the number of probiotic *Lactobacilli* species [18]. Due to its good dissolving properties, ethanol can enhance the absorption of many carcinogenic compounds that occur in everyday life [19].

Importantly, tobacco compounds and alcohol show a synergistic association with HNC development. Studies reveal that tobacco and alcohol users are significantly more likely to develop HNSCC than non-drinkers and non-smokers (even 14 times more likely among heavy smokers and drinkers) [4,20]. As mentioned above, ethanol is a good solvent that facilitates the absorption of tobacco-derived carcinogens by increasing mucosal permeability and consequently enhancing their negative impact [18,19,24].

Additionally, both tobacco and alcohol are sources of acetaldehyde; therefore, simultaneous use of those substances results in greater exposure and significantly increases their mutagenic effect [22]. Moreover, toxins present in alcohol and tobacco generate reactive oxygen species and chronic inflammation, thereby amplifying synergistic adverse effects contributing to carcinogenesis [18,19,25].

Viral infections also promote HNC. Human papillomavirus is the most common virus contributing to HNC. HPV occurs in the form of over 200 genotypes, divided into 5 subgroups [26]. Three of them, α (mucosal), β, and γ (cutaneous) HPV, influence the oral cavity. α-HPV, especially HPV-16, is related to HNC [26,27,28]. One study shows that α-HPV infection increased the HNC risk 4.6 times compared to the control group, and HPV-16 made the likelihood of HNC occurrence 16 times greater (and 22.6 times greater after adjustments) [26]. HPV-related (HPV+) HNC affects younger demographics, mainly males with higher socioeconomic status, than HNC related to other factors (HPV-) [28,29]. Additionally, HPV+ HNC shows a better survival rate than HPV- HNC [29]. While HPV- HNC contributes to the HNC formation by disrupting the DNA structure due to the action of carcinogenic compounds, the HPV+ HNC induction mechanism is mainly related to the action of two viral oncogenes, E6 and E7 [18,27]. After infecting stratified squamous epithelium cells, the viral genome undergoes its first replication and is divided into two cells- a basal cell and a keratinocyte, where it replicates at a higher number and contributes to the enhanced expression of protein (e.g., E6 and E7) [28,29]. E7 degrades pRb, leaving E2F transcription factors unbound, which activates S-phase genes and ultimately leads to unregulated cell cycle progression. In turn, E6 protein binds to E3 ubiquitin ligase (E6AP), resulting in p53 degradation, preventing damaged cells from apoptosis and allowing their proliferation. Those mechanisms contribute to carcinogenesis [18,28,29]. Other viral infections also demonstrate an impact on HNC formation. Importantly, besides HPV, there are also other viruses contributing to HNC induction, including Epstein–Barr virus (EBV), hepatitis B virus (HBV), hepatitis C virus (HCV), and human immunodeficiency virus (HIV) [30,31,32,33,34].

In addition to the risk factors mentioned above, there are more factors contributing to HNC development, such as betel quid chewing [4,35], gastroesophageal reflux disease [36], radiation [37], genetic predispositions [38], and epigenetic changes [39]. Other determinants are suspected to reveal the carcinogenic effect on HNC formation (e.g., asbestos, opium), but there is not enough data to confirm this effect [40]. However, one of the postulated mechanisms triggering HNC is cellular senescence.

## 3. Cellular Senescence

**Figure 2:**
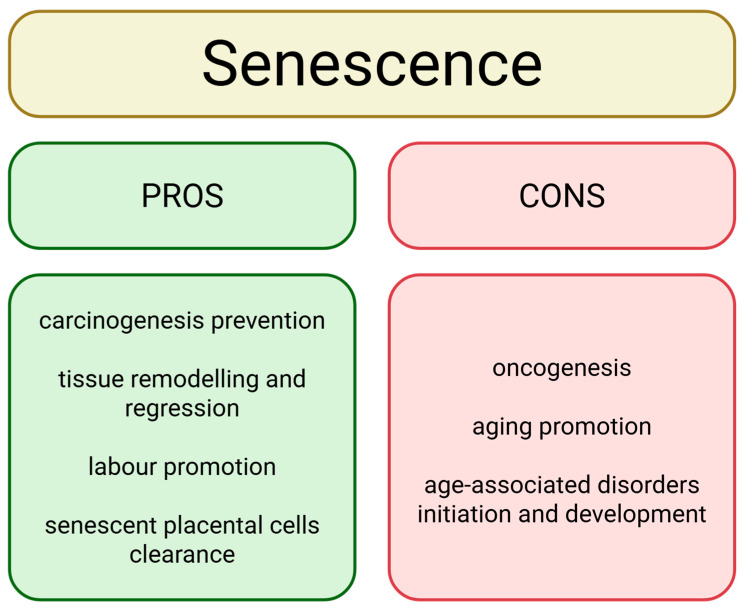
Overview of the positive and negative effects of senescence. Senescence plays an important protective role, but excessive accumulation of senescent cells and SASP (senescence-associated secretory phenotype) can result in adverse effects. Created in BioRender. Rubis, B. (2025) https://BioRender.com/2lgnjan (accessed on 12 December 2025).

Cellular senescence is a biological process caused by stress, including a variety of damaging factors [41,42]. Senescence involves the stable arrest of the cell cycle and, consequently, the cessation of cell growth and division [42]. The characteristic features of senescence occur at several levels, including the genetic/epigenetic, mRNA, and protein levels. It can involve various molecular mechanisms such as chronic DNA damage response activation (e.g., p53 activation), cyclin-dependent kinase inhibitors engagement (e.g., p16), secretory phenotype alteration, antiapoptotic genes upregulation (e.g., BCL-2), metabolism dysfunction, and endoplasmic reticulum stress [41,42]. The complexity of the pathways of this process is responsible for the cell’s structural abnormalities [41]. Senescence can be induced by multiple factors, including telomere shortening or erosion, DNA damage (by radiation, drugs, oxidation), oncogene activation or tumor suppressor inactivation, mitochondrial dysfunction, DNA methylases or histone deacetylases inhibitors, chromatin disorganisation, fibrosis, and primary senescent cells induction [41,43,44]. On the one hand, undisrupted and well-controlled senescence represents an important biological process by playing an important role in preventing cancer formation, tissue remodeling and regeneration, and promoting wound healing. However, it usually changes with aging or due to response to excessive cellular stress. This is especially while uncontrolled and accelerated senescent cells accumulation can lead to oncogenesis and accelerate aging and development of age-associated diseases (Figure 2) [41,45,46,47,48]. Senescent cells secrete various factors, including extracellular matrix components, proteases, cytokines, chemokines, and growth factors, called senescence-associated secretory phenotype (SASP) [43,44]. SASP affects cell, tissue, and organ morphology [49]. It alters the cells via paracrine and endocrine signaling, affecting cells in the immediate surrounding and further cells, leading to organ architecture and infrastructure change, such as fibrotic tissue reorganization [43,45,49]. The SASP factors also play an important role in activating immune cell response, recruiting macrophages, natural killer cells, neutrophils, and CD4 T cells. Consequently, senescent cells can be identified and removed from the organism, thereby contributing to the prevention of cancer formation and other diseases primarily associated with aging [43,49]. However, excessive SASP activity leads to an overactive immune response and chronic inflammation, called inflammaging. The constant immune cell response and elevated levels of pro-inflammatory factors negatively impact nearby and distant cells. The affected cells cause further inflammation, amplifying the impact on the microenvironment, creating the positive feedback loop [43,45,49]. Additionally, SASP can contribute to tumor formation by promoting angiogenesis [43]. Although SASP plays a major role in carcinogenesis, senescence can also promote cancer formation via senescence-associated reprogramming. Cells require certain conditions to maintain their senescent state; without them, they can re-enter the cell cycle. Specific mutations, such as loss of p16 expression, p53 or Rb inactivation, or Myc overexpression, can cause escape from senescence. While in senescence, cells undergo metabolic and epigenetic changes and accumulate DNA damage, making them potentially malignant. Once they resume proliferation, these altered cells can lead to carcinogenesis [50]. Altogether, mutation in p53 (TP53) disrupts its normal tumor-suppressing role, which usually involves triggering cell cycle arrest, DNA repair, or senescence in response to stress like DNA damage. However, mutant p53 can actually promote cancer progression by allowing cells to bypass senescence and even drive metastasis, while sometimes paradoxically inducing a detrimental, pro-tumorigenic senescence (SASP) in tumors, especially after chemotherapy, leading to resistance and relapse [51,52]. Therefore, the complex role of mutated p53 in cancer development remains ambiguous.

Chemotherapy induces senescence in B-cell leukemia cells. During the senescent state, these cells undergo reprogramming, including upregulation of stem-cell pathways such as Wnt signaling. After escaping senescence, they exhibit enhanced carcinogenic potential and greater aggressiveness than before entering the senescent state, demonstrating the pro-tumorigenic role of senescence [53]. In metabolic-dysfunction-associated steatohepatitis, senescence induced by metabolic stress via p53 contributes to upregulation of fructose-1,6-bisphosphatase 1 (FBP1). AKT and NRF2 activation caused by the metabolic dysfunction leads to FBP1 and p53 suppression. This results in uncontrolled proliferation of previously senescent cells and ultimately tumorigenesis [54].

**Figure 3:**
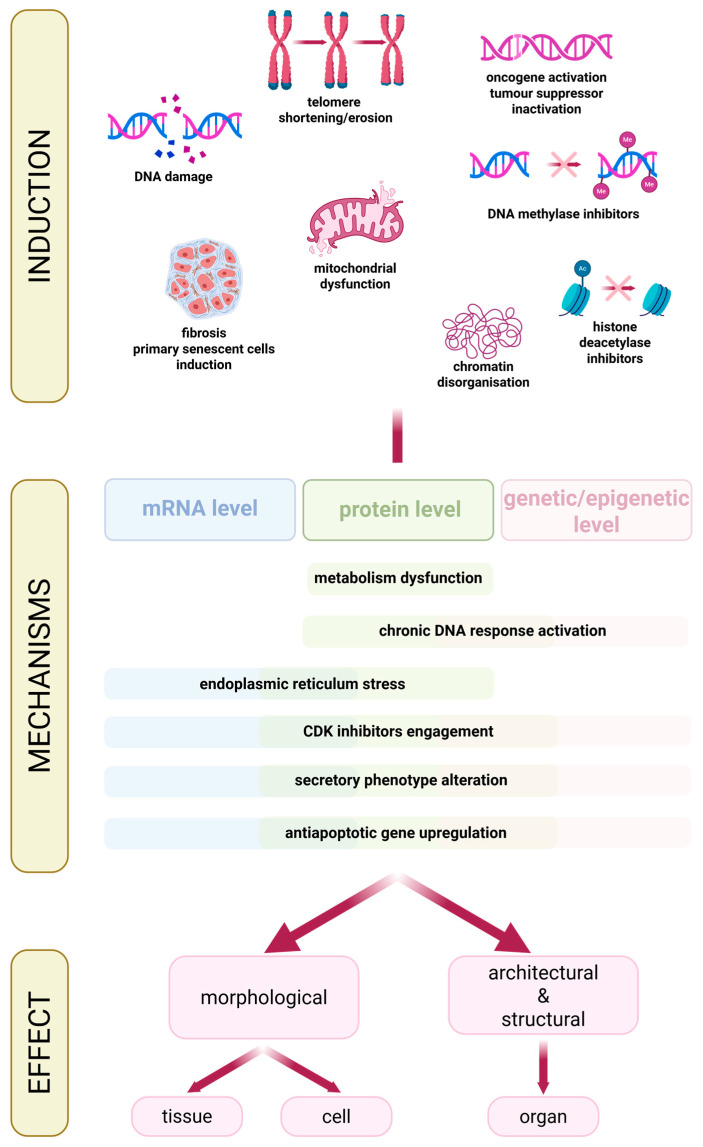
Induction, mechanisms, and effects associated with senescence. The figure illustrates the main causes of senescence induction; molecular mechanisms at the mRNA, genetic/epigenetic, and protein levels; and the impact on cells, tissues, and organs. CDK—cyclin-dependent kinase. Created in BioRender. Rubis, B. (2025) https://BioRender.com/2lgnjan (accessed on 12 December 2025).

As we know, several types of senescence have been reported so far. Cellular senescence can be categorized according to induction factors. The main types include replicative senescence (caused by telomere shortening during consecutive cell divisions). Stress-induced premature senescence is due to various stressors, such as ROS, irradiation, or DNA damage. Other types are oncogene-induced senescence (in potentially malignant cells) and therapy-induced senescence (resulting from different types of cancer treatment, including chemo-, radio-, and targeted therapy) (Figure 3) [3,55,56,57,58]. In recent years, cellular senescence has gained extensive attention and is widely researched in oncology. The senescence process engages tumor suppressors, such as RB (retinoblastoma) protein and regulatory pathways that simultaneously play a role in carcinogenesis, including p16 and p53/p21 signaling pathways [59]. While acute stress causes early phase senescence induction, influencing the p53/p21 signaling pathway, chronic stress affects p16 (cell cycle inhibitor) and is responsible for maintaining senescence [59].

## 4. Senescence in Head and Neck Cancer

**Figure 4:**
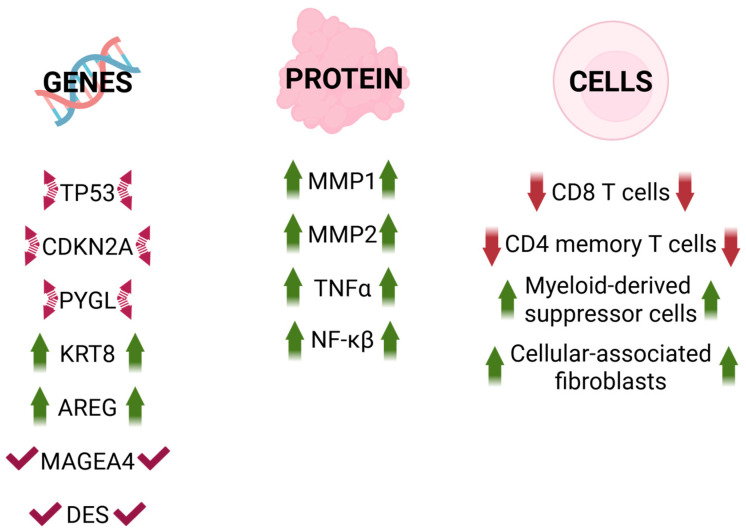
Main senescence-associated alterations and mutations in genes, proteins, and cells with significance in head and neck cancer (HNC) prognosis and development. Created in BioRender. Rubis, B. (2025) https://BioRender.com/2lgnjan (accessed on 12 December 2025).

The subject of senescence in head and neck cancer has not been broadly researched yet. However, there are some studies [60] regarding molecular pathways involved in senescence-related HNC development. This concept corresponds to the mechanisms found in other cancer types (Figure 4).

In general, oral keratinocytes and fibroblasts secrete SASP, which results in genomic instability and inflammation, contributing to OSCC formation. Mechanisms involved in this process include such SASP-related pathways as NF-κB, mTOR, STAT, and IL-6 [61]. Upregulation of SASP caused by radiotherapy was also linked to poorer prognosis in HNC [62].

A study conducted on samples derived from HNSCC patients showed increased levels of four selected SASP factors, namely, IL6, IL1β, CXCL1, and TNF-α, in tumor tissues compared to normal tissues [63]. IL6 is also present in other types of cancer, including ovarian, cervical, breast, and bladder cancers [64,65,66]. SASP factor upregulation is related to poor survival in patients with HNSCC [67]. The level of MMP1 is increased in HNSCC according to The Cancer Genome Atlas (TCGA) database, resulting in a lower survival rate [68]. Based on the same database, HNSCC samples are characterized by high mutation frequency in TP53 and CDKN2A genes, and a higher mutation level of the TP53 gene is correlated with higher cancer aggressiveness [69,70]. Those two genes are also associated with pancreatic ductal adenocarcinoma and are a risk factor in papillary thyroid cancer [71,72,73]. Moreover, senescent fibroblasts secrete VEGF, which is correlated with tumor development and angiogenesis [69]. Additionally, bioinformatics analysis of HNSCC samples from the TCGA database identified five cellular senescence genes (PYGL, KRT8, AREG, MAGEA4, DES) involved in cancer-related pathways contributing to increased risk in cancer prognosis [70] Those genes were reported to

- PYGL: glycolysis-related gene that is significantly upregulated in HNSCC. Its expression correlates with overall survival in HNC patients, and it can act as an independent factor for HNSCC prognosis. Its expression decreases in senescent cells [70,74].
- KRT8 (Keratin 8): primary component of the intermediate filament cytoskeleton mainly in simple epithelial tissues. It is frequently dysregulated during cancer progression and metastasis. Overexpression enhances cell proliferation and migration in gastric and lung cancers, whereas reduced expression significantly inhibits cell proliferation, migration, and epithelial–mesenchymal transition (EMT). Additionally, it is identified as a pan-cancer early biomarker [70,75,76,77].
- AREG (Amphiregulin): epidermal growth factor receptor (EGFR) ligand. It plays a critical role in several aspects of cancerogenesis, including cancer cell growth, invasion, metastasis, angiogenesis, and resistance to apoptosis. It is considered a critical component of the signaling pathways that drive the induction of senescence [70,78,79].
- MAGEA4: a member of the melanoma-associated antigen (MAGE) family, highly expressed in various tumor tissues but exhibiting low levels in normal tissues (excluding testis and placenta). High expression of MAGEA4 is associated with poor outcomes in cancer [80].
- DES (Desmin): muscle-specific protein that serves as a key structural component in cardiac, skeletal, and smooth muscle cells. It is used as a myoblast marker and in studies monitoring the progression of cellular senescence [81,82].

All these genes were indicated as potential senescence markers with negative implications and poor prognosis [70].

Poor prognosis is also related to TNF-α and NF-κB upregulation [62,83]. In vitro studies demonstrated IL8-mediated activation of STAT3, leading to higher MMP-1 expression in fibroblasts and, consequently, tumor progression and increased migration ability [68]. Additionally, MMP-2 from senescent fibroblasts also contributes to cancer invasion and dis-cohesion of keratinocytes in genetically unstable oral squamous cell carcinomas (GU-OSCC) [84,85]. One of the senescence-inducing factors in GU-OSCC fibroblasts is the ROS transfer from keratinocytes, which also correlates with TGF-β [86,87]. Importantly, keratinocytes might play an important role in the interaction, as these cells are generally known to be affected by senescence processes [88]. Overall, CAFs secrete various factors, which are highly related to HNSCC and OSCC tumorigenesis and treatment outcomes [89]. Cells with higher senescent score are characterized by higher levels of M2 macrophage cells associated with HNSCC development [70,90]. Senescent score is also a significant prognostic factor in HNSCC development, and its increased levels are correlated with worse overall survival [70]. Telomere DDR can regulate SASP, and short telomere length is associated with HNC and oral cancers [91,92]. Expression of five SRlncRNA genes is also associated with HNSCC formation [93].

## 5. Current Senescence-Associated Cancer Treatments

**Figure 5:**
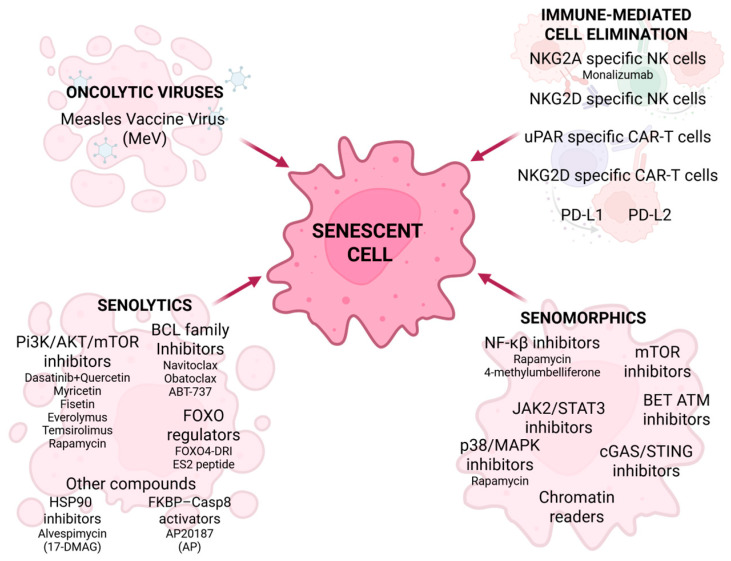
Senotherapy in cancer treatment. Senotherapeutic strategies include the use of senolytics, senomorphics, oncolytic viruses, and immune-mediated cell elimination, along with their respective targets of action in various types of cancer. Created in BioRender. Rubis, B. (2025) https://BioRender.com/2lgnjan (accessed on 12 December 2025).

Senotherapy is a relatively new approach in cancer treatment. The aim of senotherapy is to eliminate senescent cells or inhibit their growth. Cells that express and secrete SASP are responsible for aging-associated disability and diseases, including cancer. There are three main categories of aiming senolytic cells: senescent cell removal, SASP inhibition or modification, and immune-mediated senescent cell elimination (Figure 5) [94,95]. Senolytics are a group of drugs responsible for the removal of senescent cells, resulting in SASP alleviation [94]. They can be divided into four groups: PI3K/AKT/mTOR inhibitors, BCL family inhibitors, FOXO regulators, and other compounds [96,97,98]. Dasatinib inhibits tyrosine kinase and interferes with platelet-derived growth factor, contributing to inhibition of cellular growth and apoptosis progression [96,98,99,100]. Quercetin, a naturally derived flavonoid, inhibits the activity of the PI3K/AKT/mTOR pathway, promotes a senolytic effect in senescent human endothelial cells, and shows an apoptotic influence on breast cancer lines and ovarian cancer cells [96,98,99,101]. Various studies show the senolytic properties of dasatinib and quercetin combined therapy in many diseases [99,101,102], but there is little research in the cancer field. This combination was the first senescence-targeted approach to show results in SASP reduction and induction of cancer cell apoptosis. With radiation therapy, D + Q showed an anti-melanoma effect in a mouse model, and with carboplatin it reduced ovarian cancer metastasis [96,98,102]. Other senolytic flavonoids are myricetin and fisetin [96,99,100,102,103].

In contrast to senolytics, senomorphics reduce SASP but do not eliminate senescent cells [96,97,103]. They inhibit many SASP-associated cellular pathways, including NF-κB, JAK2/STAT3, mTOR, p38/MAPK, cGAS/STING, BET ATM, and chromatin readers [44,97,102]. Rapamycin affects p38/MAPK, inhibiting breast cancer. JAK2/STAT3 inhibitors led to antitumor immune response in PTEN-deficient senescent prostate tumors [97]. Anticancer properties were also exhibited after BET and STING inhibition [102]. Another type of senescence-focused therapy targets the immune system. The approaches include using natural killer (NK) cells, uPAR-specific CAR-T cells, NKG2D-specific CAR-T cells, PD-L1, and PD-L2 [97,98,103]. NK cell-mediated senescent cells elimination was exhibited in cancer cell models, which suggests that NK cell-based therapies (e.g., adoptive NK cell transfer or therapeutic antibodies) may contribute to alleviation of cellular senescence in cancer [97]. NKG2A and NKG2D are receptors found on the NK cell surface. Targeting NKG2A and enhancing NK cell activity induces senolysis, which may suggest employing therapeutic antibodies targeting NKG2A, such as monalizumab, in cancer treatment [97]. NKG2D is a receptor found on both NK and CAR-T cells. The overexpression of histocompatibility antigen alpha chain E (HLA-E) results in upregulation of NKG2D, leading to senescence-related bypass of immune response [97,98]. The research shows that therapy with NKG2D-CAR T cells reduces senescence, which suggests its future application in age-related diseases and cancer [97,98]. Urokinase-type plasminogen activator receptor (uPAR) is a specific marker of senescent cells. The uPAR-specific CAR-T cells can eliminate senescent lung adenocarcinoma in mice [97,98,103]. PD-L1 and PD-L2 immune checkpoint receptors are overexpressed in senescent cells [97,98,103]. Two clinical trials, the KEYNOTE-158 and CHECKMATE-358, exhibit positive results in PD-1 targeted cervical cancer in the advanced stage [98]. Oncolytic viruses are a novel strategy used in cancer treatment. They act not only by eliminating non-senescent cancer cells but also by removing the senescent cells. Measles vaccine virus (MeV) demonstrates senolytic properties against cancer cells [99].

**Figure 6:**
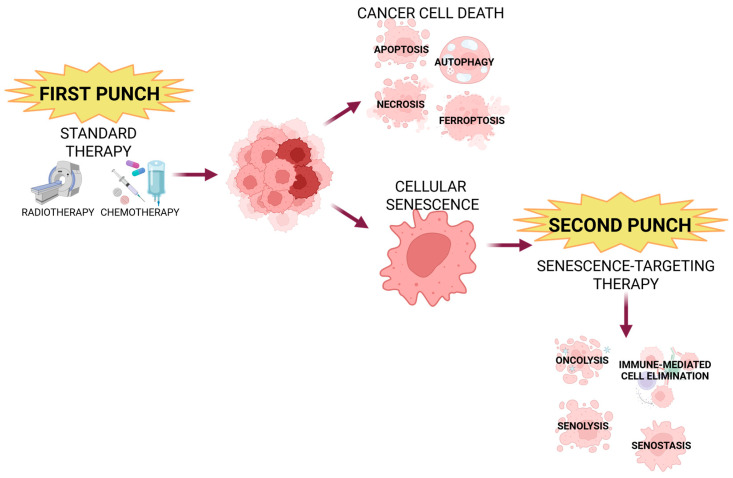
One-two punch strategy in cancer treatment. First step of this novel approach involves standard therapy (such as radio- or chemotherapy), followed by senotherapy to eliminate senescent cells created and/or survived after the first phase. Created in BioRender. Rubis, B. (2025) https://BioRender.com/2lgnjan (accessed on 12 December 2025).

There is a new approach in the field of anticancer therapies called the “one-two punch” strategy (Figure 6) [96,97,99,102,104]. Firstly, patients are treated with standard, pro-senescence therapies like chemo- or radiotherapy, followed by targeting senescent cells [44,96,97,99,102,104]. The first step leads to cancer cell death, but not all cells can be eliminated, so there is also induction of senescence; then, those senescent cells are eliminated in the second step [96,99]. There are numerous examples of preclinical studies employing this technique. For example, a combination of XL413, CDC7 kinase inhibitor, and mTOR inhibitor AZD8055 exhibits positive results targeting hepatocellular carcinoma. Yet another mTOR inhibitor, temsirolimus, together with docetaxel, shows effectiveness in prostate cancer [96,102]. Other senogenic–senolytic combinations with anticancer results are Olaparib and Navitoclax, a BCL-2 inhibitor (ovarian cancer); Gembicatine and Digoxin, MEK/CDK 4 inhibitor and CAR-T cells, CDK 4/6 + MEK inhibitors; and anti-PD-1 (pancreas cancer), Doxorubicin, and anti-PD-1 (breast cancer) [96,97,99,102]. The “one-two punch” cancer strategy is a promising approach but still needs verification and optimization, especially since it shows some limitations referring to tumor heterogeneity (different cancer types/cells), drugs bioavailability, overcoming the tumor’s microenvironment barriers (including SASP), specificity towards tumor cells, and resistance mechanisms.

Studies concerning senolytic therapy in head and neck cancer are still very limited. In the in vitro study, two flavonols, fisetin and kaempferol, induced apoptosis in human tongue squamous cell and submandibular gland cancer lines through BCL-2 inhibition and caspase-3 activation [105]. Piperlongumine is another natural compound triggering cellular apoptosis in HNC and OSCC in vitro [106,107]. Conventional HNC radio- and chemotherapy leads to epigenetic age acceleration (EAA) and senescence induction [108]. For example, CDK4/6 inhibitors used for cancer therapy increase the senescence of the tumor cells and SASP level in the microenvironment [105]. Therefore, most of the studies involving senescence in HNC therapy focus on a two-step approach. Navitoclax, a BCL-2 family inhibitor used after senescence-induced treatment with Palbociclib (CDK4/6 inhibitor), cisplatin, and 4-methylumbelliferone, eliminates senescent cells in HPV HNSCC, human and murine HNC, and OSCC cell lines, respectively [109,110,111]. Interestingly, metformin, initially used for type 2 diabetes, also exhibits a senostatic effect, and after Abemaciclib (CDK4/6 inhibitor)-induced SASP, metformin promotes apoptosis in HNSCC [104,112]. Panobinostat (BCL-XL inhibitor) removes senescent HNSCC cells after chemotherapy with Cisplatin/Taxol [96,113]. Additionally, Nivolumab, an anti-PD-1 drug combined with Sitravatinib, improves the treatment outcome in oral cancer and with Docetaxel in head and neck squamous cell cancer [114,115]. Moreover, exercises, as a part of prehabilitation, influence various aspects of senescence, including senescent T cells, NK cells, leukocyte telomere lengths, and neutrophils, which leads to a delay in immunosenescence onset [116].

The elimination-based strategy for senescent cells could be efficient in HNC treatment. Conventional therapies help eliminate cancer, but at the same time, they facilitate the senescence increase, which hinders complete recovery and may contribute to disease recurrence. Senescence-targeted therapy plays an important role in decreasing SASP-associated inflammation, a significant factor supporting tumor development.

## 6. Senotherapy in HNC-Clinical Trials, Limitations, and Future Perspectives

There are many clinical studies focused on targeting senescence. However, they focus mainly on neurodegenerative or chronic diseases such as multiple sclerosis [117], Alzheimer’s disease [118], chronic kidney disease [119], or osteoarthritis [120]. They also focus on improving the health of older people [121]. Recently, the results of a clinical trial involving senolytics in HNSCC (COIS-01) have been published. The researchers combined anti-PD-1 therapy with senolytics (dasatinib + quercetin), which confirmed high efficacy (33.3% significant pathological response rate) and enhanced safety (4.2% adverse events) of the treatment [122,123].

As mentioned above, in the clinical trial, the number of side effects was low [123]. However, some limitations and risks must be taken into consideration when discussing senotherapy. Senescent cells, when at an optimal level, play a beneficial role. They enhance cellular reprogramming, which boosts tissue repair and optimizes the wound-healing process, preventing excessive scarring formation [124,125] and facilitating muscle regeneration [126]. They also participate in embryonic development [127]. While in excess, senescent cells have a pro-tumorigenic role, their appropriate level, however, has an opposite effect—it protects against the development of cancer [128,129]. This suggests that utilizing senotherapeutics in one aspect may negatively influence other mechanisms contributing to adverse effects.

Additionally, there are some limitations regarding assessing the senescence levels in samples. There is no single biomarker that clearly confirms senescence and can be applied to all sample types. Due to the complex nature of senescence, there are many biomarkers utilized in senescence, including senescence-associated β-galactosidase [130], histone γ-H2AX double-strand DNA break [131], Sudan Black B [132], cytosolic double-stranded DNA [133], DNA methylation entropy [134], cyclin-dependent kinase inhibitors (p16Ink4a and p21Cip1/Waf1) [135], nuclear accumulation of globular actin [136], heterochromatin loss [137], and telomere shortening [138]. The most common technique, SA-β-gal staining, can be used in vivo and in cryopreserved tissues; however, it does not yield satisfactory results with paraffin-embedded samples [139,140]. Moreover, one study showed that not all of the cell types express SA-β-gal activity, as it comes from the GLB1 gene, which encodes lysosomal β-D-galactosidase. Cells without functional GLB1 can become senescent but will not express SA-β-gal [141]. Sudan Black B staining addresses the issue of tissue preservation and can be performed on formalin-fixed, archived samples; however, it cannot be used independently, as it detects lipofuscin pigment granules that are present not only in senescent cells but also in deteriorated cells [132]. The second most common biomarker, telomere shortening, should also be utilized in combination with other biomarkers, due to the fact that telomere length varies between tissues and organisms. Combining that biomarker with histone γ-H2AX or SA-β-gal activity staining would provide a more reliable assessment [142]. Taking into consideration all existing limitations, researchers are continually searching for new senescence biomarkers that could conclusively verify the presence of senescence.

Future perspectives associated with senescence and senotherapy in cancer treatment are very promising. Using multiple markers to detect and identify senescence can help integrate suitable senolytics into the antitumor treatment regimen [63,143]. Additionally, understanding the role of particular senescent cells in the HNC development may indicate the choice between senescent cells removal or SASP reduction. Moreover, considering the non-specific nature of general senolytics, there should be more studies concerning targeted senolytic therapies, such as cell/protein-specific substances or utilization of delivery systems [144,145]. Further studies should include epigenetic aspects and environmental conditions, and due to the diversity of the risk factors, it may be a real challenge to find efficient prevention and/or therapeutic strategies.

## 7. Conclusions

Many factors, including alcohol, tobacco, and HPV infection, support head and neck cancer development. Some of them facilitate the progression of cellular senescence, which contributes to cancer development by affecting many molecular pathways. There has been a significant increase in focus on senescence in carcinogenesis, and numerous studies have investigated senescence-associated treatments for various types of cancer. The results of those studies indicate an excellent potential for antitumor therapies involving senescence that indicates senotherapy as a potential element of an adjuvant cancer therapy. However, the senescence in the HNC field still remains understudied. Because the senescence-related signaling pathways involved in oncogenesis overlap in different types of cancers, the results of the previously mentioned studies suggest the application of senescence-targeting therapies in HNC as well, leaving room for further research.

We illustrated how senescence could shift from a protective to a pro-tumorigenic and harmful process. To simplify: induced senescence → chronic inflammation → carcinogenesis. This pathway could be blocked/attenuated by senolytics involvement. So, due to high variability of the factors that are capable of induction of senescence (including radiotherapy, oxidative stress, and oncogene activation), this process can be perceived as a non-direct but critical factor in carcinogenesis, not only in HNC. However, HNC development is associated with smoking and alcohol consumption, and both factors contribute to induced senescence-associated reprogramming. Thus, mechanistic studies indicate the link between environmental factors, senescence induction, and carcinogenesis. Altogether, senotherapy shows huge potential as an element of an adjuvant cancer therapy.

## Data Availability

No new data were created or analyzed in this study. Data sharing is not applicable to this article.
